# ISG15 induction is required during L1-mediated colon cancer progression and metastasis

**DOI:** 10.18632/oncotarget.27390

**Published:** 2019-12-24

**Authors:** Sanith Cheriyamundath, Sayon Basu, Gal Haase, Harry Doernberg, Nancy Gavert, Thomas Brabletz, Avri Ben-Ze’ev

**Affiliations:** ^1^ Department of Molecular Cell Biology, Weizmann Institute of Science, Rehovot 76100, Israel; ^2^ Experimental Medicine I, Nikolaus-Fiebiger-Center for Molecular Medicine, University of Erlangen-Nuernberg, Erlangen 91054, Germany

**Keywords:** ISG15, L1, colorectal cancer, metastasis

## Abstract

Hyperactivation of Wnt/β-catenin target gene expression is a hallmark of colorectal cancer (CRC) development. We identified L1-CAM (L1) and Nr-CAM, members of the immunoglobulin family of nerve cell adhesion receptors, as target genes of the Wnt/β-catenin pathway in CRC cells. L1 overexpression in CRC cells enhances their motile and tumorigenic capacity and promotes liver metastasis. L1 is often localized at the invasive edge of CRC tissue. Using gene arrays and proteomic analyses we identified downstream signaling pathways and targets of L1-mediated signaling. Here, we found that the expression of interferon-stimulated gene 15 (ISG15) that operates much like ubiquitin (is conjugated to proteins by ISGylation), is elevated in the conditioned medium and in CRC cells overexpressing L1. Suppression of endogenous ISG15 levels in L1-expressing cells blocked the increased proliferative, motile, tumorigenic and liver metastatic capacities of CRC cells. ISG15 overexpression, on its own, could enhance these properties in CRC cells, but only to a much lower extent compared to L1. We show that NF-κB signaling is involved in the L1-mediated increase in ISG15, since blocking the NF-κB pathway abolished the induction of ISG15 by L1. Point mutations in the L1 ectodomain that interfere with its binding to L1 ligands, also inhibited the increase in ISG15. We detected high levels of ISG15 in human CRC tissue cells and in the adjacent stroma, but not in the normal mucosa. The results suggest that ISG15 is involved in L1-mediated CRC development and is a potential target for CRC therapy.

## INTRODUCTION

Colorectal cancer (CRC) is the second most common cause of death related to cancer in the world. A hallmark of CRC development is an aberrant activation of the Wnt/β-catenin pathway, already at an early stage of the disease, resulting in hyperactivation of β-catenin-TCF target gene expression [1–3]. Overt activation of this pathway is also required at later stages of cancer development, and involves the induction of target genes that regulate cancer cell invasion and metastasis. We identified Nr-CAM and L1, members of the immunoglobulin-like neuronal cell adhesion receptors, as target genes of the Wnt/β-catenin pathway in CRC cells [2, 3] and detected L1 at the invasive front of human CRC tissue [4]. Overexpression of L1 in CRC cells confers increased proliferation in low serum, enhanced motility, tumorigenesis and liver metastasis [5]. Using gene microarray and proteomic analyses, we identified a number of genes and signaling pathways induced by L1 overexpression in CRC cells and validated their relevance to CRC development [6–12]. In this study, using a proteomic analysis of proteins whose level is increased in the secretome of L1-overexpressing CRC cells, we found that the ubiquitin-like interferon-induced gene 15 (ISG15) [13], is one of the proteins whose expression is dramatically increased in L1-overexpressing CRC cells. Here, we determined the key role of ISG15 in the tumor promoting properties conferred by L1 in human CRC cells and tissue.

## RESULTS

### ISG15 expression is induced in the secretome and in CRC cells overexpressing L1

**Figure 1:**
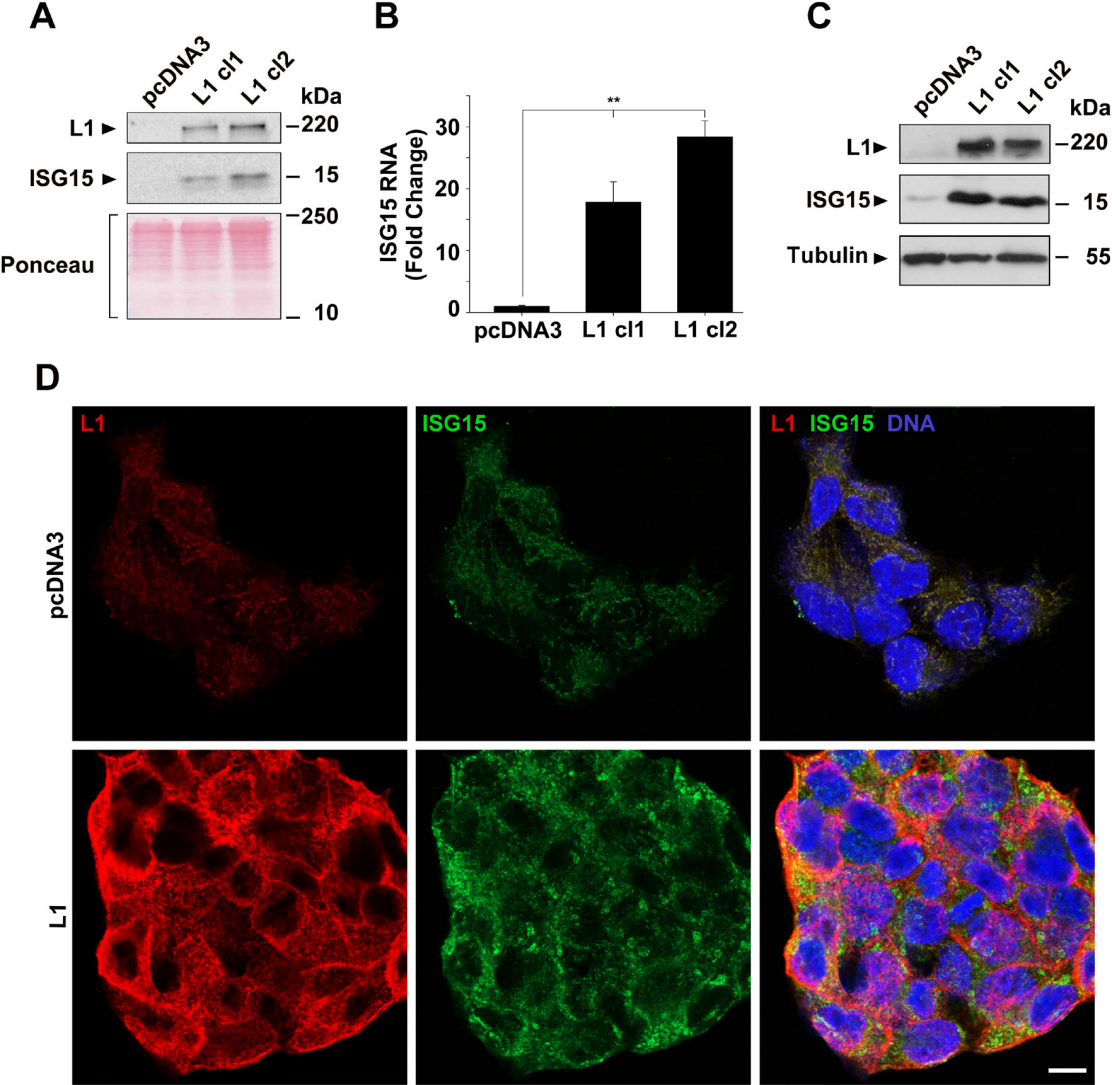
Induction of ISG15 expression by L1 in CRC cells. (**A**) The culture medium from an equal number of LS 174T cells expressing the pcDNA3 control plasmid and clones of cells stably expressing L1 (L1 cl1 and cl2), that were kept for 2 days in serum-free medium, was analyzed for the presence of ISG15 by western blotting. (**B**) The expression of ISG15 RNA was determined by qRT-PCR in the cell clones described in (A). (**C**) Western blot of the cell clones described in (A) for ISG15 in the cell layer. Ponceau staining was used for determining equal loading and quality control of the western blots. (**D**) Immunostaining of LS 174T cells stably expressing the pcDNA3 plasmid, or L1, with antibodies to ISG15 (green), L1 (red) and DAPI (blue). The bar represents 10 μm.

We wished to determine the proteins and signaling pathways that are involved in L1-mediated progression of colorectal cancer (CRC). We employed the LS 174T human CRC cell line stably overexpressing L1 and compared the proteins secreted into the conditioned medium of these cells using mass spectrometry [12], to those of cells transfected with the control pcDNA3 plasmid. Among the proteins in the culture medium whose levels were most dramatically induced in L1-overexpressing CRC cells, we identified the ubiquitin-like interferon-stimulated protein of 15 kDa (ISG15, Supplementary Table 1). Western blot analysis confirmed the increase in ISG15 in the conditioned medium of LS 174T cell clones overexpressing L1 (Figure 1A). Moreover, an elevation in ISG15 RNA (Figure 1B) and protein (Figure 1C) was also observed in the cell layer of LS 174T cell clones overexpressing L1. Immunofluorescence analysis of ISG15 localization in cells overexpressing L1 revealed an increase in endogenous ISG15 staining, mostly in a granular pattern in the cell cortex of L1-overexpressing cells, while control CRC cells displayed only a very weak staining for ISG15 (Figure 1D). These results suggest that L1 overexpression in CRC cells results in increased expression and secretion (into the culture medium) of endogenous ISG15.

### Modulation of ISG15 levels in CRC cells affects cell proliferation, motility and tumorigenesis

**Figure 2:**
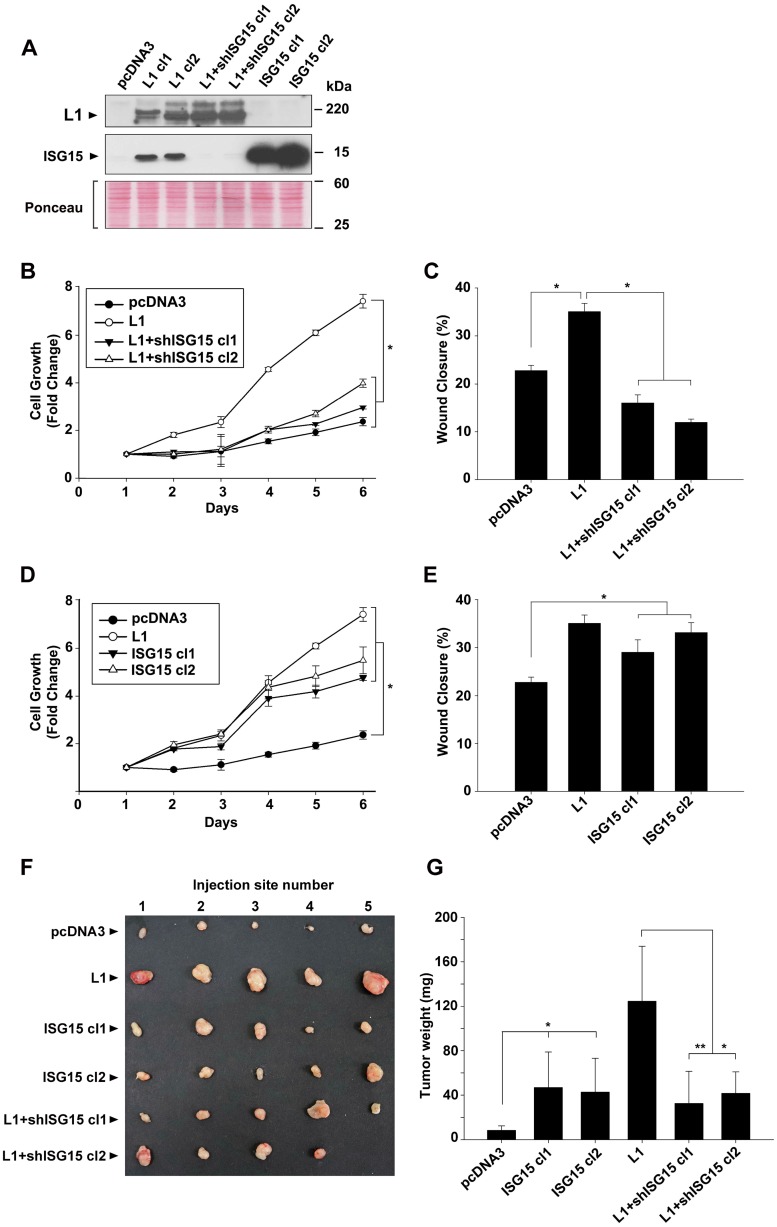
Modulation of ISG15 expression in CRC cells affects cell proliferation, motility and tumorigenesis in human CRC cells. (**A**) Individual LS 174T cell clones stably expressing the control plasmid (pcDNA3), L1, L1+shRNA to ISG15 (L1+shISG15 cl1 and cl2) and ISG15 (ISG15 cl1 and cl2) were isolated and the expression of the various proteins was determined by western immunoblotting with the relevant antibodies. (**B** and **D**) The proliferation of the cell clones described in (A) was determined under stress (in the absence of serum) during 6 days in culture. (**C** and **E**) The motile properties of the cell cones described in (A) were determined by the “scratch” wound closure method, 24 hours after introducing the wound in confluent monolayers. (**F**) The tumorigenic capacity of the cell cones described in (A) was determined by injecting 1.5 × 10^6^ cells s. c into groups of 5 mice, per each cell line, into immunodeficient mice and the tumors formed were excised after 2 weeks and photographed and their weight was determined (**G**).

To determine the requirement for increased ISG15 in the tumorigenic properties conferred by elevated L1 expression, we isolated CRC cell clones overexpressing L1 in which the levels of endogenous ISG15 were suppressed using shRNA to ISG15 (Figure 2A, L1+ISG15 cl1 and cl2). The proliferation rate under stress (in the absence of serum) of such CRC cell clones in which ISG levels were suppressed was reduced to that of control CRC cells not expressing L1 (Figure 2B). In addition, the motile abilities of these cells determined by the “scratch wound” closing experiment were also reduced, even below that of control pcDNA3-transfected CRC cells (Figure 2C), implying that ISG15 plays an important role in the motility of CRC cells.

We wished to determine to what extent increased ISG15 expression, on its own, can confer the increase in the proliferative and motile capacities of CRC cells. For this, we isolated LS 174T cell clones overexpressing ISG15 (Figure 2A, ISG15 cl1 and cl2). The results summarized in Figure 2D and 2E demonstrate that ISG15 overexpression in CRC cells, similar to L1, could elevate the proliferation of cells under stress (in the absence of serum) (Figure 2D) and an increase in the motility of cells in the “scratch wound” experiment (Figure 2E), but to a lesser extent than L1 overexpression. Finally, we have analyzed the tumorigenic ability of these different CRC cell clones (in which ISG15 levels were manipulated) *in vivo* by s. c injection into immunocompromised mice (Figure 2F and 2G). The results showed that ISG15-overexpressing cells displayed an increase in tumorigenic capacity compared to control CRC cells, but to a lesser extent than L1 overexpression (Figure 2G). The L1-mediated increase in tumorigenesis required an elevation in ISG15 since suppression of ISG15 levels dramatically decreased the tumorigenic capacity of L1 in CRC cells (Figure 2G, compare L1 to L1+shISG15 cl1 and cl2). We concluded that the elevated expression of ISG15 is necessary for the L1-mediated increase in the proliferation, motility and tumorigenesis of CRC cells.

### An elevation in ISG15 is required for the L1-mediated metastasis of CRC cells to the liver

**Figure 3:**
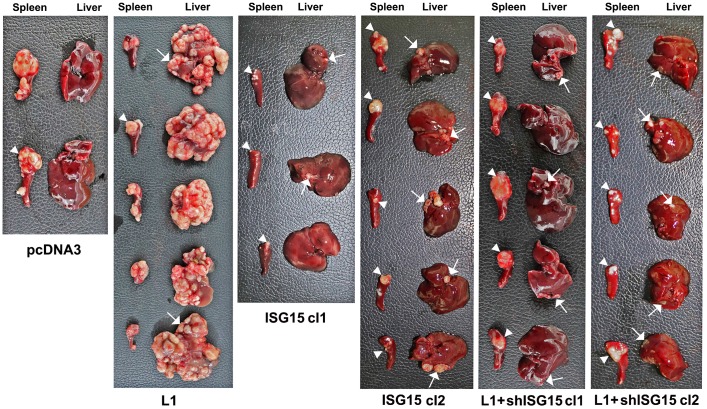
Overexpression of ISG15 enhances liver metastasis of CRC cells and ISG15 suppression in L1-overexpressing cells blocks metastasis. Immunodeficient mice were injected into the tip of the spleen with 1.5 × 10^6^ cells of the CRC cell clones described in Figure 2A and development of tumors at the site of injection (in the spleen) and metastasis in the liver were determined after 6 weeks. The spleens and livers were excised and photographed and quantitative analysis of metastasis formation is described in Supplementary Figure 1.

The liver is the preferred organ in human CRC metastasis. In previous studies, we have shown that L1 overexpression in CRC cells confers liver metastasis in a mouse experimental model [5]. We wished to determine whether the increase in ISG15 during L1-mediated CRC development is necessary for liver metastasis. Immunocompromised mice were injected into their spleen with the CRC cell clones described in Figure 2A and the development of liver metastases was determined. The results summarized in Figure 3 and Supplementary Figure 1 show that while LS 174T CRC cells do not form liver metastases (Figure 3, pcDNA3), as previously demonstrated [5], L1-overexpressing cells completely filled the liver with metastatic foci (Figure 3, L1). Unlike CRC cells overexpressing L1, ISG15-overexpressing CRC cells only formed a low number of small metastatic foci in the liver (Figure 3, ISG15 cl1 and cl2). The increase in ISG15 in L1-overexpressing cells was necessary for liver metastasis since suppression of ISG15 levels in such cells dramatically reduced their metastatic ability (Figure 3, L1+shISG15 cl1 and cl2). In all cases, the cells proliferated at varying degrees at the site of injection (in the spleen), but as we previously reported, there was no correlation between tumor cell proliferation in the spleen and the metastatic capacity to the liver of these cells [5]. Taken together, these results suggest that the increase in ISG15 is a necessary step in L1-mediated metastasis of CRC cells to the liver.

### Point mutations in the L1 ectodomain and inhibition of NF-κB signaling abolish the increase in ISG15 by L1 expression and the ISGylation of proteins

**Figure 4:**
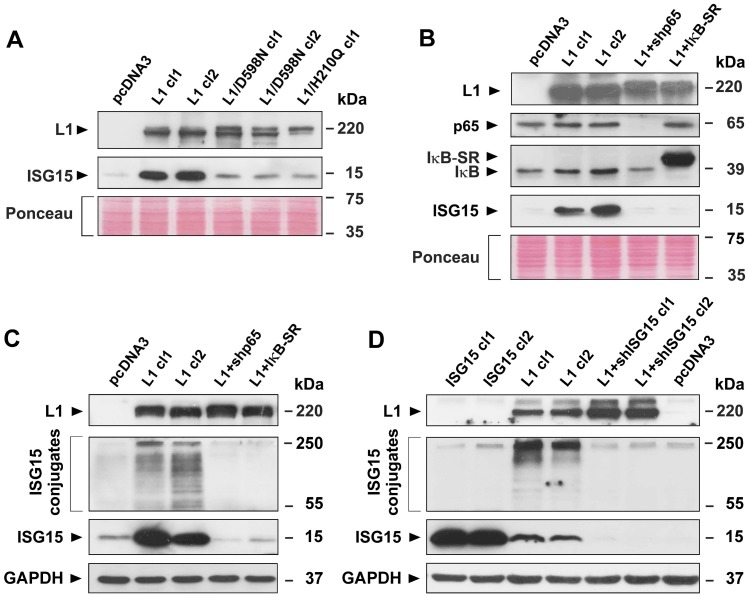
Induction of ISG15 and ISGylation by L1 in CRC cells is blocked when NF-κB signaling is inhibited, or when point mutant L1 forms are expressed in cells. (**A**) LS 174T CRC cell clones stably expressing the pcDNA3 control plasmid, or L1 (L1 cl1 and cl2), or the mutant forms of L1 (L1/D598N cl1 and cl2 and L1/H210Q cl1) were analyzed for ISG15 expression by western immunoblotting. (**B**) CRC cell clones stably expressing the control plasmid pcDNA3, L1 and L1+shRNA to p65 (L1+shp65), or L1 and the IκBα super repressor (IκB-SR) were analyzed for the expression of p65, IκB-SR, IκB, and ISG15 with the relevant antibodies. (**C**) ISGylation and the levels of free ISG15 were determined in the CRC cell clones described in (B) by western blotting using antibodies to ISG15. (**D**) ISGylation and free ISG15 levels were determined in CRC cell clones overexpressing ISG15, L1 and L1+shRNA to ISG15. Ponceau staining and GAPDH levels served as markers for equal loading of the gels.

We wished to determine the signaling pathways involved in the L1-mediated increase in ISG15 expression that lead to enhanced tumorigenesis and metastasis. In previous studies, using point mutants in the L1 ectodomain that affect its interaction with ligands, we found that such L1 mutants lost the ability to confer increased tumorigenesis and metastasis [10]. Using clones of CRC cells expressing the L1/H210Q and the L1/D598N point mutations in the L1 ectodomain that affect L1-L1 binding (H/210Q) and the binding of L1 to ECM components (L1/D598N), we found that such CRC cells display a much-reduced level of ISG15 (Figure 4A), suggesting that an unperturbed ectodomain binding of L1 with ligands outside the cell is necessary for propagating the downstream signaling that leads to increased ISG expression.

One of the signaling pathways that we previously detected that operates downstream of L1 to bring about the increase in L1-mediated tumorigenesis and metastasis is the NF-κB pathway [6, 14]. Inhibition of the NF-κB pathway by suppressing the levels of the p65 subunit of NF-κB with shRNA to p65, or the inhibition of this signaling pathway by overexpressing the mutant IκBα super-repressor (IκB-SR) in CRC cells, both blocked the induction of ISG15 expression by L1 (Figure 4B).

ISG15 operates in the cell much like ubiquitin, forming conjugates (ISGylation) with a large number (hundreds) of proteins in the cell [15, 16]. We wished to determine whether the formation of such ISGylated conjugates is affected when the levels of ISG15 are suppressed by shRNA to ISG15, or when the NF-κB pathway is inhibited. The results summarized in Figure 4C demonstrate that the suppression of ISG15 levels in human CRC cells, or the inhibition of NF-κB signaling, both reduced not only the free pool of ISG15, but also the extent of ISG15 conjugates formation, suggesting that increased ISGylation in L1-overexpressing cells might be required for the promotion of CRC progression. The uniqueness of the induction of L1-mediated ISGylation is demonstrated in Figure 4D by showing that ISG15 overexpression in CRC cells does not result in increased ISGylation (Figure 4C, ISG15 cl1 and cl2), while suppression of endogenous ISG15 expression in L1-overexpressing cells eliminates both ISGylation and the effects on tumorigenesis (Figure 2G) and metastasis (Figure 3) conferred by L1.

### ISG15 expression is induced in both CRC cells and in the stroma but is absent in normal mucosa

**Figure 5:**
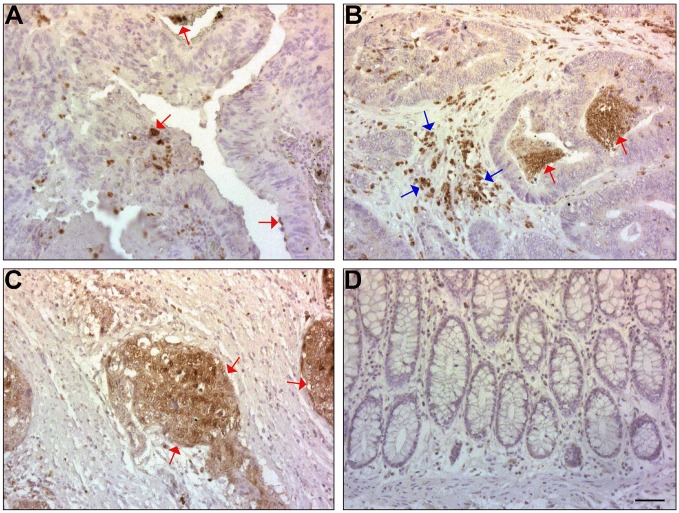
ISG15 expression is induced in CRC tissue and in the adjacent stroma, but not in normal mucosa of the colon. (**A**) ISG15 was immunolocalized in single cells and in small clusters of CRC cells in the tumor tissue in 51% of the samples (red arrows). (**B**) ISG15 was also detected in 44% of the CRC cases in the lumen (red arrows) of the colonic tumors and in the stroma (blue arrows). (**C**) ISG15 was detected in the whole tumor tissue in poorly differentiated areas of some cases (red arrows). (**D**) The normal colonic mucosa was not stained with antibody to ISG15. The bar represents 200 μm.

We wished to determine the localization of ISG15 expression in human CRC tissue. Thirty-nine human CRC tissue samples were analyzed by immunohistochemistry with antibodies to ISG15 (Figure 5). We observed the staining of individual tumor cells in 51% of the cases (20 of 39) for ISG15 (Figure 5A, red arrows). Strong staining of the tumor stroma was also observed in 51% (20 of 39) of the cases (Figure 5B, blue arrows), together with staining of detached tumor cells in the colonic lumen in 44% of the cases (17 of 39) (Figure 5B, red arrows). Strong immunostaining of the entire tumor tissue for ISG15 was observed in poorly differentiated areas in 3 of 39 of the cases (Figure 5C, red arrows), while the normal mucosa did not display significant staining for ISG15 (Figure 5D). These results suggest that ISG expression is elevated in both CRC tumor tissue and in the stroma and thus might play a significant role in CRC development.

## DISCUSSION

In this study, we showed that among the proteins whose levels are most dramatically elevated by L1-overexpression in CRC cells is the ubiquitin-like interferon induced gene 15 (ISG15). We detected a significant increase in ISG15 both in the secretome and in cultured human CRC cells overexpressing L1. The increase in ISG15 was required for the pro-tumorigenic and metastatic properties involving increased cell proliferation under stress, enhanced cell motility and metastasis to the liver that are conferred by L1-expression in CRC cells. Interference with L1-mediated signaling by point mutations in the L1-ectodomain that are known to affect its adhesive properties and abolish its tumorigenic and metastatic properties [10], and which cause serious brain developmental diseases in patients [17, 18], also eliminated the ability of L1 to induce ISG15 and to confer enhanced tumorigenesis and metastasis. We found that the mechanism/s downstream of L1 that bring about the increase in ISG15 involve NF-κB signaling, since blocking this pathway by various methods eliminated the ability of L1 to induce ISG15. Increased NF-κB signaling and elevated ISG15 expression were recently observed also in BRCA1 mutants of fallopian tube epithelial cells [19] and in ovarian cancer cells [20]. In addition, cRel, a subunit of NF-κB, was shown to bind and activate the *ISG15* gene promoter, supporting the involvement of NF-κB in regulating the transcription of the *ISG15* gene [21].

ISG15 is suggested to operate much like ubiquitin and brings about the conjugation (ISGylation) of ISG15 to a wide variety of proteins (between 100–300 proteins) [15, 16] and both pro-tumorigenic and tumor suppressive roles have been suggested for increased ISG15 expression in various types of cancer [22–25]. Because of the large number of potential ISG15 target proteins and the conflicting results regarding its pro-tumorigenic and anti-tumorigenic potential, the roles of ISG15 and ISGylation in tumorigenesis still remain to be elucidated.

In addition to its conjugation to target proteins, ISG15 was detected as an unconjugated protein, and in its free form ISG15 was suggested to function as a cytokine and as an intracellular interacting partner, acting independently of its conjugation to a target protein [13]. In our study, we detected ISG15 both in a free, secreted fraction and also as a free component in the cytoplasm, as well as in conjugation with a large number of proteins. Interference with L1-mediated signaling that resulted in the suppression of ISG15 expression and which blocked the increase in tumorigenesis and metastasis, also inhibited ISGylation, suggesting that the two processes are related. Finally, we detected increased ISG15 expression in human CRC tissue in tumor cells and in the adjacent stroma, while the normal mucosa did not display detectable ISG15 staining. Therefore, while the exact mechanism/s by which increased ISG15 expression confers tumorigenesis and metastasis in CRC has yet to be discovered, the current study suggests that blocking the increase in ISG15 could be applied as an effective approach to the therapy of CRC metastasis.

## MATERIALS AND METHODS

### Cell culture, transfection, cell proliferation and motility assays

The cell line LS 174T was grown in RPMI medium-1640 (Gibco, UK) containing 10% FBS (Gibco, Brazil) and penicillin/streptomycin solution (Biological Industries, Israel). LS 174T-L1, LS 174T-ISG15, LS 174T-L1/D598N and LS 174T-L1/H210Q cells [10] were maintained in RPMI medium-1640 containing neomycin (800 μg/ml). LS 174T-L1+shISG15, LS 174T IκB-SR and LS 174T-L1+shp65 cells [6, 14] were maintained in RPMI medium-1640 containing both neomycin (800 μg/ml) and puromycin (10 μg/ml) [7, 10]. Transfection of LS 174T cells was performed using Lipofectamine™ 2000 (ThermoFisher Scientific, MA, USA) according to the manufacturer’s instructions. For cell proliferation assays, 2,000 cells were seeded in 96-well plates in medium containing 0.1% FBS and the proliferation rate was determined by the XTT assay kit (Biological Industries, Israel). Cell motility assay was assessed by the artificial “scratch wound” closure assay as described [6].

### Plasmids

The ISG15 expression vector was obtained from Dr. Y. Song (Chinese Academy of Medical Sciences and Peking Union Medical College, China). ShRNA to ISG15 was prepared using pSUPER. puro by following the manufacturers protocol (pSUPER. puro RNAi System, OligoEngine, WA, USA). The target sequences used for the preparation are described in Supplementary Table 2.

### Immunoblotting and immunofluorescence

Antibodies used for immunoblotting were: rabbit anti-L1 (a gift from Dr. V. Lemmon, University of Miami, FL, USA) at 1:8,000 dilution, mouse anti-ISG15, sc-166755 (Santa Cruz Biotechnology, Inc. USA) diluted 1:1,000, rabbit anti-phospho-IκBα #2859 (Cell Signaling Technologies Inc., MA, USA) diluted 1:1,000, rabbit anti-NF-κB p65, sc-109 (Santa Cruz Biotechnology, Inc. USA) diluted 1:1,000, mouse anti-GAPDH, sc 47724 (Santa Cruz Biotechnology, Inc. USA) diluted 1:2,000, mouse anti-β-tubulin (Sigma-Aldrich, MO, USA) diluted at 1:5,000. Cells were lysed in RIPA buffer [10].

Western blots were developed using the ECL method (Amersham Biosciences, UK). To analyze proteins secreted into the cell culture medium, cells were grown until confluence and the medium was replaced with medium without FBS for 24 hours. The medium was collected and centrifuged at 4,500 rpm for 10 minutes to sediment cell debris and the supernatant was filtered through a 0.22 μm syringe filter. The filtered medium was mixed with cold ethanol at a 3:1 ratio and with NaCl (5 M, 40 μl per 1 ml medium) and incubated overnight at ‒20°C and the denatured proteins were precipitated by centrifuging at 15,000 RPM for 30 minutes. Protein levels in cell lysates were determined by the BCA assay and the precipitated proteins were suspended in Laemmli’s SDS-PAGE loading buffer.

For immunofluorescence, cells cultured on glass coverslips were permeabilized with 0.5% Triton X-100 in 4% PFA for 2 minutes [12]. The primary antibodies against L1 and ISG15 were the same ones used for immunofluorescence and for immunoblotting. The secondary antibodies were Alexa Fluor 488-labeled goat anti-mouse IgG (ABCAM, Cambridge, UK) diluted 1:1,000, Cy3-labeled goat anti-rabbit IgG (Jackson Immunoresearch laboratories, PA, USA) diluted 1:1,000. Nuclei were stained using 5 μg/ml 4′-6-diamidino-2-phenylindole (DAPI, Sigma-Aldrich, MO, USA). Images were acquired using the Zeiss LSM 800 confocal microscope, equipped with the Zeiss objectives 40×/1.3 NA and ZEN imaging software (Carl Zeiss Microscopy GmbH, Jena, Germany) and analyzed using the Image J and FIJI software.

### Quantitative RT-PCR

Total RNA was isolated from cells using the Bio-Tri reagent (Bio-Lab, Israel) according to the manufacturer’s protocol. First strand cDNA was synthesized using the SuperScript™ II Reverse Transcriptase (ThermoFisher Scientific, MA, USA). The ISG15 primers used were, forward: 5′-GAGAGGCAGCGAACTCATCT, reverse: 5′-AGCATCTTCACCGTCAGGTC. Data analysis was performed employing the ΔΔCT method with the StepOne software v2.3 (ThermoFisher Scientific, MA, USA).

### Mass spectrometry

The mass spectrometry analysis of the medium collected from LS 174T and LS 174T-L1 cells was performed at the proteomic unit of the Weizmann Institute of Science, as previously described [12].

### Tumor growth and metastasis assays

Subcutaneous tumor growth was induced as described [6]. Briefly, 1 × 10^6^ cells were injected into 5 different sites in the flanks of male nude mice [11]. The ability of cells to metastasize was determined by injecting 1.5 × 10^6^ cells in 20 μl PBS into the distal tip of the spleen of 6 weeks-old male nude mice as described [5]. Mice were anesthetized by peritoneal injection of 1 μl/mg xylazine (20 mg/ml) and 1 μl/mg ketamine (100 mg/ml). After 7 weeks, the animals were sacrificed, and primary tumor formation in the spleen and metastasis appearance in the liver were examined. The tumor area was calculated using the ImageJ software.

### Ethics approval

The Ethics Committee of the Weizmann Institutional Animal Care and Use (IACUC) reviewed, approved and supervised the animal studies (Permission number 11530219-2).

### Immunohistochemistry

Immunohistochemistry was carried out on 39 paraffin-embedded human colorectal adenocarcinomas using a rabbit anti ISG15 antiserum (antibodies-online #ABIN2844984), diluted 1:150, as previously described [10].

### Statistical analysis

In the mouse metastasis experiments, statistical significance between the pcDNA3 and ISG15 cell clones was determined by the Wilcoxon Signed-rank test. The significance in the differences between L1 and L1+shISG15 cell clones was determined using the Mann-Whitney test. The significance of all other comparisons was determined by ANOVA. A *P* value of < 0.05 was considered significant

## SUPPLEMENTARY MATERIALS



## Abbreviations

L1: L1 cell adhesion molecule
CRC: colorectal cancer
ISG15: interferon-stimulated gene 15
TCF: T-cell factor
NF-κB: nuclear factor kappa B
IκBα: inhibitor of kappa B alpha
