# Supplementary material for: ISG15 induction is required during L1-mediated colon cancer progression and metastasis
Source: Oncotarget. 2019 Dec 24;10(67):7122–31. doi: 10.18632/oncotarget.27390 (PMC6935256; doi:10.18632/oncotarget.27390)
Supplement: Supplementary file 1 [file oncotarget-10-7122-s001.pdf]

ISG15 induction is required during L1-mediated colon cancer progression and metastasis

SUPPLEMENTARY MATERIALS

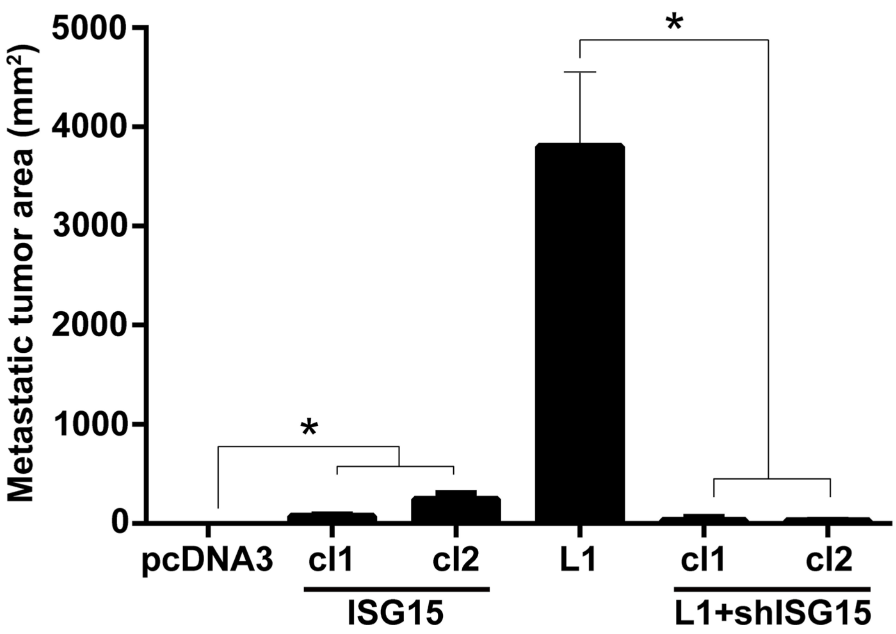

Supplementary Figure 1: Suppression of CRC metastatic ability by inhibiting ISG15 expression. The metastatic tumor area in the liver was determined in nude mice injected in the spleen with the human CRC cell line (LS 174T) stably transfected with pcDNA3, ISG15 cl1 and cl2, L1, L1+ shISG15 cl1 and cl2.

Supplementary Table 1: Proteins secreted at higher level from LS 174T cells expressing L1 compared to control LS 174T cells

Gene symbol	Description	Fold change
EPS8L3	Epidermal growth factor receptor kinase substrate 8-like protein 3	584.5
STXBP2	Syntaxin-binding protein 2	475.9
ISG15	Ubiquitin-like protein ISG15	231
SMARCE1	SWI/SNF-related matrix-associated actin-dependent regulator of chromatin subfamily E member 1	209.1
SNX5	Sorting nexin-5	104.3
TRIP6	Thyroid receptor-interacting protein 6	100.6
PARVA	Alpha-parvin	84.6
PODXL2	Podocalyxin-like protein 2	82.3
IRF2BP2	Interferon regulatory factor 2-binding protein 2	71.83
PELP1	Proline-, glutamic acid- and leucine-rich protein 1	38.3
MST1	Hepatocyte growth factor-like protein	28.3
LPP	Lipoma-preferred partner	25.2
MAP7	Ensconsin	24.8
OCLN	Occludin	22.9
ROCK2	Rho-associated protein kinase 2	20.9
CDCP1	CUB domain-containing protein 1	18.8
VCAN	Versican core protein	18.1
ACTR1B	Beta-centractin	14.5

Mass spectrometric analysis of proteins secreted into the cell culture medium from LS 174T cells stably expressing L1 compared to LS 174T cells transfected with pcDNA3. Proteins whose level was increased >10 fold in L1-expressing cells are shown.

Supplementary Table 2: Sequences of shRNA targeted against ISG15 RNA

Name	Primer sequence
shISG15-1	GATCCCCCGGAAATAAAGGCTGTTGTTTCAAGAGAACAACAGCC TTTATTTCCGTTTTTA
shISG15-2	GATCCCCGCGCAGATCACCCAGAAGATTCAAGAGATCTTCTGGG TGATCTGCGCTTTTTA
shISG15-3	GATCCCCCTGAGCATCCTGGTGAGGATTCAAGAGATCCTCACCA GGATGCTCAGTTTTTA
